# From uprising to secession: a plea for a localized and processual approach to the avatars of the yellow vest movement

**DOI:** 10.1057/s41253-022-00198-6

**Published:** 2022-10-26

**Authors:** Olivier Fillieule, Alexandre Dafflon, Zakaria Bendali, Maite Beramendi, Davide Morselli

**Affiliations:** 1 UNIL-CRAPUL, Lausanne, Switzerland; 2 UNIL-LIVES, Lausanne, Switzerland

**Keywords:** France, Yellow vests, Online mobilizing, Social media, Survey research, Mixed methods

## Abstract

For about fifteen years now, so-called leaderless movements, often stemming from appeals via social networks and having recourse to lasting or episodic occupations of public spaces have flourished. This has had the effect of calling into question the usual toolkit used by social movement scholars to study them. More precisely, it is a question of which levels, dimensions and units of analysis are relevant, especially when it comes to describe movements’ social base and worldviews. In this paper, based on a localized and a long-term collective undertaking, begun from the very beginning of the Yellow Vests movement in November 2018, we discuss those shortcomings and suggest avenues for analysis. We rely on three sets of data in a concomitant manner: life history calendars, social media data (mainly Facebook) and field research (participant observation and biographical interviews). By considering time and place as key variables, we offer an innovative way to describe how the YVs movement has been constructed in a constant flux of change.

## Introduction

The past decade has seen an unexpected resurgence of street-level protest movements around the world, from the uprisings of the Arab Spring to the rise of Indignados in Spain, from the mushrooming of Occupy movements to the outburst of the Yellow Vests (thereafter YV) in France. Often stemming from appeals via social networks and having recourse to lasting or episodic occupations of public spaces, these movements are also polycentric, loosely organized and leaderless (Tüfekçi 2017).

Faced with this new type of mobilization, researchers find themselves in the same situation as Mr. Palomar, Italo Calvino’s character (1986) who undertakes to “know” his meadow but gives up, because he cannot rationally decide where the meadow begins and ends, what are the grasses that make up the meadow, to the exclusion of the weeds, and at what scale he should ideally situate himself in order to embrace with his gaze this “infinite meadow.”

There is no doubt about it: the usual toolkit used by social movement scholars to study them is here seriously challenged, especially when it comes to describing movements’ social base and worldviews. First, surveys by questionnaires come up more than ever against the absence of a reference population, making any sampling strategy quite hazardous; second, when in classical social movements ideologies and claims were considered as prerequisites, here worldviews are emerging at the same time as the mobilization, in a constant flow during the course of the action. Hence the necessity to find new ways of constructing our object of study and of studying it through innovative instruments. This paper is an attempt in this direction, by proposing an original articulation between quantitative and qualitative data, based on a localized and longitudinal analysis.

The following analysis is based on a localized and a long-term collective undertaking, begun from the very beginning of the YV movement in France. The movement originates in a Facebook group launched in January 2018 and took off in November of the same year. Since then, a multitude of protest events were counted, either in the form of occupying and blocking roundabouts, or in street demonstrations. This movement was noteworthy in its duration, its territorial scope and its occurrence in urban, suburban and rural areas, as well as its blunt refusal of all leadership. It sprang from calls on the Internet, with no external support. Hence the key role Facebook has played in the birth and subsequent organization of the movement, which makes it necessary to study it in the same way as non-virtual places of mobilization. Protests have spread all over the French territory, giving rise to riotous scenes, unprecedented in the police and judicial repression they triggered.

Despite its size and scope, the YV movement came up against the intransigence of the government, which relied on three levers to crush them: a campaign of systematic denigration of the movement with the unanimous support of the major national media; fierce police and judicial repression; a major detour operation by organizing a “great national debate” which, from mid-December 2018 to mid-March 2019, allowed it to regain control of the political agenda. The decline of the movement then accelerates, marked by a decrease in the weekly number of demonstrators, the disappearance of most of the occupied roundabouts and a support of the opinion that passes below 50% of the population.

Considering the polycentric character of the movement, we decided to reduce the focus to two contrasted areas: Var department in South-East France; and a suburban area in the far Northern outskirts of Paris.^1^ In this article, for reasons of practicality, we limit ourselves to exploring some of the data collected in Var. We have chosen to study this department first of all because it was one of the high places of mobilization in the country, with emblematic traffic circles like at Cannet-des Maures, where a triumphal arch, an Eiffel tower and a Louvre pyramid were erected, attracting a lot of media attention. Another reason refers to the high scores that the extreme right has achieved there for a long time (several municipalities are held by National Front elected officials), but also because of its economic and social fabric. A semi-rural area with about 1 million inhabitants and a density of 180 people per square kilometer, Var's economy is mainly oriented towards the production of goods and services for people living in the area, whether tourists or residents. The development of the “silver economy” is very present in the department where the number of retired people is very high, resulting in a growth of home care services. The population is concentrated along the coastline and the main roads that cross the department from east to west. It is along these routes that the occupations of roundabouts have settled, aggregating people living in both urban and rural areas.

We start with a first section that reviews literature on the way movements' social base, their ideology, and the worldviews they convey have been studied methodologically, but also how those questions have been dealt with so far when it comes to the YV movement. In a second section, we describe our research design and the way we propose to combine quantitative and qualitative data, based on a localized and longitudinal analysis. To do this we articulate three sets of data in a concomitant manner: life history calendars (LHC), social media data (mainly Facebook) and field research (observations and repeated biographical interviews). In a third section, we argue the value added by such an innovative combination of data and methodological tools to describe how the YV movement is constructed in a constant flux of change. Finally, we conclude by returning to the rather homogenizing labels that have been attached to the YVs to propose some alternative paths, based on the results presented.

## Literature review

### Who are those people in the streets?

If there is a question that is both central and relatively disputed by social movement scholars, it is the definition of what a “social movement” is. It is directly linked to the question of which levels, dimensions and units of analysis are relevant to the analysis of a movement.

Thus, some researchers generalize findings from studies of local movement groups, specific action repertoires or so-called critical moments, while others draw a general picture of a movement by highlighting its supposedly most salient features and thus writing off the real diversity and complexity of the observed phenomenon. A problem of oversimplification that arises in relation to each of the general dimensions that make up a movement: its social base, its ideology and the worldviews it conveys, its objectives and the tactics it uses to achieve them, its territorial implantation and its organizational structure. (McCarthy et al. 1991).

Taking into account a movement’s social base and worldviews has proved to be the most difficult, as Social Movement Studies (SMS) have historically neglected the micro-sociological perspective because protesters were dismissed as irrational. From the 1970s onwards, social movements were understood as rational actors that balanced the costs and benefits of collective action, taking into consideration the resources available and the negative and positive incentives to political participation (McCarthy and Zald 1977). This strain of research, called resource mobilization theory (RMT) also introduced macro-contextual explanatory dimensions conceptualized as “political opportunity structures” (Tarrow 1989). This paradigm is still predominant, and is further reinforced by the application of methods such as organizational surveys or protest events analysis (Hutter 2014 for a review).

The use of qualitative methods such as in-depth interviews and life stories (Della Porta 2014) provides a deeper understanding of social movement participants. However, the problem of limited generalization remains.

Within the field of political participation studies, there have been attempts to understand social movements (conceptualized herein as unconventional political participation), by means of studying political attitudes towards protest through general opinion polls in several Western countries (Barnes et al. 1979; Sajak and Haunss 2020). However, these general surveys deal with low population sub-samples that struggle to explore and explain in-group variation. These large surveys are often also more generic in nature, thus “isolating the participant from his or her environment” (Fillieule and Blanchard 2005).

In SMS, survey research and INdividual SUrveys in RAllies (thereafter INSURA) are the two main methods to describe the sociography of social movement participants, in addition to their opinions, attitudes and worldviews. Survey research is a frequently employed method in SMS (Crist and McCarthy 1996; Klandermans and Smith 2002; Andretta and Della Porta 2014) whose underlying assumption is that socio-economic living conditions determine individuals’ practices and beliefs (e.g. Cotgrove and Duff 1980 on “middle class radicalism”). The aforementioned RMT and the theory of “political opportunity structures” is at the basis of this perspective, presuming that participation is made possible by “biographical availability” (McAdam 1988), social network ties (Diani and McAdam 2003), and ideological compatibility (McAdam 1988; Snow et al. 1986). By means of control groups that distinguish between bystanders, supporters, and activists, survey research can further determine participation and non-participation. However, such studies often lack a proper sampling frame (lists of individuals or organizations that comprise the research population) and a standard of comparison (see Klandermans and Oegema 1987; McAdam 1988; Fillieule et al. 2018 for some exceptions). The exact same problems arise when it comes to research on online activism, especially since the lion’s share of this research focuses on structural and organizational aspects of social media in protest (Bennett and Segerberg 2012; Mattoni and Pavan 2018; Mattoni 2019; Schumann and Klein 2015; Tüfekçi 2017) and on the link between offline and online protest (Freelon et al. 2018; Jost et al. 2018; Ramaciotti Morales et al. 2022).

Sampling is made even more difficult by the fluctuating nature of contemporary movements, with their lack of formal boundaries and leadership, loose structure, and polycentrism. The alter-globalization movement serves as a prime example as it rendered any attempts to carry out traditional individual surveys obsolete. Hence the idea of starting rather from the observation of the events themselves, understood as collective performances which, at a given moment in time, reveal networks more or less structured around organizations, activists’ affiliations but also shared or controversial meanings.

INSURA (see Fillieule and Blanchard 2010 for a review) emerged as particularly suited for that object of study. In effect, one remembers the public debate that emerged about how to measure and assess the heterogeneity of the movement in terms of organizations as well as constituencies. To answer all these questions, the INSURA seemed an appropriate tool.

In INSURA, usual sampling strategies are impossible to use. In protest events, only some people are affiliated to organizations, and the number of organizations makes impossible any proximate to the research population. Hence, one has to use a probabilistic method, that is to say, to guarantee that all possible participants would have equal opportunity of being interviewed. To achieve that, one must take into account the fact that participants’ spatial and temporal distribution is never aleatoric. (Fillieule 1997; Favre et al. 1997) Other relevant questions, with implications for the representativeness of the sample, concern the status of the specific surveyed demonstrations vis-à-vis the social movement to be investigated since “a crowd can’t be considered as equal to a social movement constituency. Its heterogeneity is far more important and different in nature” (Fillieule and Blanchard 2010). Hence, it makes little sense to admit that social movement participation can be epitomized in a one-shot participation, especially in the case of movements which are marked by a “secular, inclusive and non-totalizing approach” and “tolerant identities” (Della Porta 2004), as opposed to the organizational identities of the past. Finally, INSURA can only capture the image of a crowd at one point in time and in one specific location, forbidding to generalize from a one-shot survey as well as making the aggregation of different surveys conducted at different times and places very unconvincing.

### Who are the yellow vests?

Studies of the YV movement have generally focused on the socio-economic attributes of participants or on their world views and political claims. Here, we briefly summarize findings before advancing a critique of the studies’ methodological underpinnings.

Surveys that are based on Facebook or INSURA data underline that the YV participants come from diverse social backgrounds, but have modest incomes and a position at the threshold of poverty or among the impoverished. Blavier (2021), for example, finds that the YVs struggle to cover what he calls constrained expenses and to pay for leisure activities and other types of goods. Research also highlights that sentiments of economic deterioration are shared subjectively by the YVs. This continuous process of socio-economic downgrading is key to understanding the YVs eruption, some authors claim. However, it remains to be explained how, under the embrace of this dominated position, these categories that are usually perceived as having divergent interests (e.g. salaried people against independent workers) have come to fight alongside each other.

In a second strain of research, worldviews and political claims have been at the center of attention. The movement is divided on subjects such as immigration and the refugee crisis, and its participants’ political ideology spans from the far left to the far right. Many refuse to position themselves on the political axis altogether (Ramaciotti Morales et al. 2021; Quantité Critique 2018). However, the political profile of the movement largely varies between studies.

An undercurrent in the study of the YVs political claims has given particular attention to the populist dimension of the movement (Rouban 2019; Tarragoni 2019), arguing that its cohesion is based on the conceptualization of “two homogeneous and antagonistic groups, ‘the pure people’ versus ‘the corrupt elite’” (Mudde 2004, 543). By using a survey carried out in Facebook groups, Guerra et al. (2019) show that the movement is structured around a populist economic discourse defined as “producerism” (Berlet and Lyons 2020) that understands “the people” as “an authentic community of hardworking people who produce and reproduce France’s wealth” (Guerra et al. 2019, 3). Facebook users contend that they feel threatened by a “‘parasitic’ caste of unproductive ‘takers’”, a discourse surpassing the simple sentiment of being left behind (Grossman 2019) or resentment towards ethnic groups (Guerra et al. 2019, 10).

Although important, these studies all overlook three aspects that we deem necessary to understand the YV movement: time, space and the reference population of the study. Firstly, many of these surveys fail to systematically build samples of respondents that might provide an accurate picture of the movement more broadly, or specific dimensions of it. The samples can be composed of activists, first-time participants, sympathizers, bystanders, etc. There is a risk of erroneous sampling both in Facebook and in the application of INSURA. INSURA aggregates different surveys conducted at different times and places, which can be problematic in terms of generalization and renders any comparison with a national control group spurious. Secondly, most of the surveys cover the very first months of the movement but do not go beyond the spring of 2019, thus not allowing for the study of evolving dimensions of the YV movement in time and in relation to local and national contexts. Work on collective identity (Melucci 1996; Regers et al. 2008) has clearly shown that claims evolve in a constant flux of change and must be considered as a process. Due to its duration and the absence of a preexisting shared political ideology and collective identity, we can assume that both individual properties and worldviews have evolved according to the protest experience, the political responses of the government—such as police repression—the turnover of activists, and political and social events. Here, Ramaciotti Morales et al.’s (2021) analysis of topics discussed on Facebook is particularly interesting. By comparing the conversations in the FB groups over time, it shows a shift in debated topics. While discussions on immigration play an important role in the first weeks of the movement, they decline markedly before disappearing entirely, in favor of institutional reforms (RIC) and police repression. Third, existing works mostly content themselves with collecting data on the situation of people *at the time* of the production of the survey. For example, rather than collecting information on the entire residential or employment history of individuals, one collects the employment status or place of residence at the time of the survey. Finally, most surveys focus on the movement at the national level, amassing data from very diverse local contexts, making any aggregate treatment hazardous.

Qualitative studies that produce data in the flow of daily interactions in local groups help to overcome some of these shortcomings. In his ethnography of young adults in small rural towns, Coquard (2019) clearly shows the variety of rural contexts, where some areas are in economic decline while others are more attractive in terms of employment and public services. Other authors have also insisted on the specific political history of each territory and its effects on mobilization. By considering the characteristics of the YV movement as embedded in specific contexts, ethnographic studies highlight how worldviews and claims are articulated in relation to local living conditions and not necessarily to the political realm. These studies signal the existence of a common social consciousness structured around a new sense of collective identity that reduces mistrust towards “welfare recipients” or “immigrants” and creates a unified popular “us” (Coquard 2019) against the “cosmopolitan and liberal elites,” what Challier (2021) calls a “protest consciousness”. They also insist on the feeling of social injustice and a desire to moralize the economy while readjusting the public investment in the most neglected territories.

Qualitative research contextualizes quantitative findings and adds analytical complexity. However, its designs generally have two possible pitfalls: firstly, these studies rarely question the constitution of their sample, in that they do not relate their findings to the more aggregated levels of the movement, locally or nationally. Secondly, while many take time into consideration, this dimension is rarely explained and investigated as such.

In the following section, we propose methodological tools that seem promising to better control the significance of our corpus, and to better take into account the changing nature of the studied phenomenon.

## Data and methods

In order to understand how such disparate and politically inexperienced individuals come together without any pre-existing mobilization structure and mobilize in such an intense way, our investigation is based on a localized and long-term collective enterprise, which began at the beginning of the movement and will continue until December 2023. To give ourselves the means to study the social base and the worldviews of the YVs in Var, with its rythms and its avatars, we propose an innovative combination of methodological tools that might hopefully reduce the blind spots highlighted in the literature review.

### A localized and temporal analysis

First of all, we began by determining a localized activist configuration (Fillieule and Broqua 2020), i.e. the perimeter we were willing and able to study. As a first step, we aimed at reconstructing activist milieus, in order to better understand the socio-economic fabric and the local spaces in which the Var yellow vests evolve. Thanks to official statistics (INSEE), we dispose of a control group at the departmental level to compare with the demographics of our respondents. We also gathered data through leaflets and fanzines collected since November 2018, as well as a systematic content analysis of press clippings from Var-Matin, the local newspaper. This allows us to reconstruct the local chronology, the networks of alliances and conflicts amongst the YVs, and to better understand the logic behind the ways the selected local chapters, with their particular localization and neighborhoods, were occupied.

Starting from in situ observations and after having established contacts with a number of YVs who took us to visit the groups they frequented, we were able to determine a set of 6 stabilized interconnected roundabouts. (see Ravelli 2020 on “roundabout clusters”) This choice was determined by the emerging networks, discussions, and exchanges that took place between participants, rather than by an a priori judgement call. If the selected roundabouts have been in sustained interrelations as of November 2018, their links have been recomposed over time, under processes of *scissiparity* (e.g. after ideological or personal conflicts), of *absorption* (e.g. following the exhaustion of smaller, less frequented roundabouts or because of repression) and of *attraction*, mainly because of the particular aura of such or such roundabout. As a result the number of activists, therefore of occupied roundabouts and encampments, has decreased continuously, a phenomenon of attrition that never stopped.

This continuous decrease is locally due to a series of overlapping and successive factors. From the very first weeks, apart from the merely curious and the sympathizers, the first defectors were some of the extreme right activists or sympathizers, who failed to take control of either the encampments or the demonstrations. A second acceleration of defection occurs in mid-March 2019 under the effect of repression that falls on the movement at the national level, pushing the most moderate and many women to leave the movement while a more limited fringe becomes radicalized. The increasing biographical unavailability of many activists who, after several months of intense activism, were forced to reconsider their investment in favor of their professional and family activities, then reduces the circle of the most active to retired and inactive people, and to individuals living at the margins of the wage-earning sector. Finally, here and there, the presence of activists from *La France Insoumise* is becoming more visible and vocal, which contributes to the disengagement of some. At the end of the summer of 2019, only three roundabouts remain active, which does not mean, however, that all those who are no longer there have disengaged, as a significant part of them remain active on their FB groups and pages, keeping the flame alive by constituting what can be described as a “virtual abeyance structure.” (on abeyance, see Taylor 1989).

### A sampling framework that allows a collective biography

To offer a valid test of the classical hypothesis on the factors for individual activism in the movement, it would be necessary to assemble a group of respondents considered as representative of the YV population along time. However, this is impossible given the nebulous character of this movement, the continuous attrition of membership since November 2018 and the multiplicity of forms and levels of commitment. Lacking a reference population, we did put into operation an innovative method in order to provide an accurate picture of both the diversity and the common features of the YVs along time in the areas we study.

To build a stratified sampling framework and to extract potential interviewees, we initially used the FB groups and pages of our 6 local chapters. Being at a relatively small scale and in the absence of any SMO or coordination committees, our case study represents a peculiar connection between offline and online activism, in which street protesters discuss and connect through FB groups and pages, making it possible to observe the unfolding of discussions.^2^ While this source does not provide systematic sociographic information, it does allow the building of a typology of modes of commitment, according to their *timing*, *duration* and *intensity*. In particular, we were interested in sampling these respondents who are impossible to contact in street protests, notably people who stopped participating at the time of data collection.

To estimate the level of activism on FB, we selected all the users who commented or replied to any of the posts on the monitored pages and groups. This choice was dictated to distinguish between simply sharing posts (which could also be the work of bots) and actually engaging in the discussion. Our sample was therefore composed of 3055 users. Data were anonymized to guarantee users’ privacy and safety.

We thus computed a series of indicators to evaluate the level and period of activity of each user. In particular, we considered the date (in weeks from the beginning of the movement) of first and last posting activity to assess the duration of the active period. To estimate how active the user was, we calculated the average number of posts per active week. Finally, to assess the engagement in the online posting, we calculated the average number of words per post. This last measure was used to distinguish between those users who shared videos and pictures and those who actively engaged in typing text.

Thereafter, we computed Gower distance between users and used the distributed stochastic neighbor embedding (t-SNE, van der Maaten and Hinton 2008) as implemented in the Rtsne package for R (Krijthe 2015) to identify different user profiles. The appropriate number of clusters was assessed with the Silhouette method (Rousseeuw 1987).

The analysis extracted four profiles. Results reported in the table below shows that the profiles were clearly distinguished by the different periods of their online activity. Profile 1 (28% of the users) included people who were mostly active in the first period of the movement. These users were also the ones who mostly used Facebook, with 1.8 posts on average per week. Users in Profile 2 (25%) where active around the middle part of the movement and Profile 4 (20%) during the last period, when the movement started reigniting, on the eve of the opposition movement to the pension reform (December 2019). Finally, Profile 3 (27) included users who were active for most of the movement's timeline, covering a span of about 40 weeks. Interestingly, these long-lasting users were also the least active profile, though they wrote  slightly more text when engaging. The difference in the average text length is, however, minimal among all profiles (Table 1).

**Table 1:** Clustering of the Var Facebook users in Var

Variable	Profile 1 *n* = 849 (28%)	Profile 2 *n* = 776 (25%)	Profile 3 *n* = 825 (27%)	Profile 4 *n* = 605 (20%)
Week of first post	9.13	27.34	19.44	51.45
Week of last post	14.04	33.76	57.19	55.93
Avg n. posts per active week	1.81	1.27	1.07	1.34
Avg n. words per post	15.32	15.43	15.49	13.44

Although we had initially thought of using this data to extract a sample of interviewees out of the 3055 users, we finally decided against it. Firstly, because the contacts made via *Messenger* proved to be very unprofitable, especially for those who were mere sympathizers and did not become activists (a good deal of cluster 1); secondly because, contrary to general expectations, the movement continued. We could  thus maintain our presence on the field, allowing us to extend the lists of potential interviewees among activists, directly or through a “snowball” approach, hence avoiding the risk of collecting only the names of the most visible people at the various times we were present, those who remained in contact with our prior contacts, or those who had become most visible along time. This point is crucial given the succession of ruptures and splits experienced by all the local groups, but also the frequency of the quiet departures of many activists tip-toeing their way out throughout the period. All in all, we were able to collect around 600 contacts (phone numbers, messengers, sometimes only names or nicknames).

Since the local chapters studied mobilized an average of 3000 people over the entire period (activists but also mere sympathisers for whom we had no real interest), the 600 or so names of activists we collected (i.e. 20% of the estimated total) is not insignificant. What is more, thanks to FB clustering, we could systematically see in which clusters the people on our extended list were falling, which allowed us to retain a sample of interviewees in the same proportions. Based on this strategy we have extracted a group of around 200 persons from the name list, paying attention to the distribution by age, gender and the evolution of the average size of the roundabout they were mainly attached to.

Given the movement's properties, we believe we have gone as far as we can in constructing a meaningful sample on the basis of which we can confidently say something accurate about our roundabout clusters and that we can compare to the control group of the Var population.^3^

We invited those respondents to fill a life history calendar (thereafter LHC), a tool that has the advantage to amalgamate a number of different event histories in a single, large chart (Freedman et al. 1988; Glasner and van der Vaart 2009). By inviting individuals to list, in a chronological table, their residential, affective, training, professional, and activist paths since birth, we provide ourselves the means to paint a portrait of the group based on longitudinal retrospective data. (Rossier and Fillieule 2019) To date, 144 LHC and calendars of activities have been collected and coded, associated to 50 biographical interviews.^4^

### A systematic study of Facebook pages and groups

As stated before, FB has played a key role in creating a public space for discussion among YVs, an arena for expressing their demands, their anger and disgust, their hopes and dreams. It constitutes a precious, unbiased—because of the absence of post hoc reconstructions—archive of positions, emotions and attitudes of the participants along time.

Data on the activity of the 6 FB groups and pages covering the studied space are collected by means of the FB Group API, which allows to extract posting activity as well as dates and other metadata; in particular subscribers posting activity (texts or shared content and dates). Following access restriction and revision of the terms of service operated by FB in July 2019, special authorization is needed to use the API. Our interface (application in FB terms) to collect the data has been reviewed and approved by Facebook Inc. on September 3, 2019. In this study we adopted a mixed-method framework relying on computational social science techniques and in-depth qualitative analysis to investigate the content of the FB conversations. As argued by Lewis et al. (2013), a hybrid approach allows for an understanding of large-scale data, while leveraging on the strength of qualitative analysis.

To this goal our analytical approach was divided into two steps, each mixing qualitative and quantitative methods. To dig into the underlying dimensionality of the text and highlight the content of the discussions in the targeted FB groups, we jointly applied topic modeling and content analysis. In particular, we used the structural topic modeling framework with spectral initialization as implemented in the stm package for R (Roberts et al. 2014). Topic modeling is a technique that models the co-occurrence of features in language and assumes that text can simultaneously talk about several topics at the same time. It estimates the probability that each topic is present in a document of the corpus. Therefore, the goal of topic modeling is to determine the number of topics that most suitably describes a corpus while also describing the likelihood of these topics in each document of the corpus. Similar to exploratory factor analysis, a series of indicators can be used to identify the optimal number of topics, as well as their interpretability.

To investigate the development of themes over time, the evolution of identity talks' features, and their relationship with contextual events, we transformed the content of the analysis into time series which indicated the distribution of each shared topic across time and the collective identity reference within each topic. All the analyses were conducted on French text. In order to make them more accessible to the international reader, translated text has been presented in English throughout.

## A mixed- method analysis

### The social base of the Yellow vest movement in Var

In this section, based on an initial exploration of our corpus of LHC (*N* = 144), we explore the question of how class, defined in occupational terms, (Erikson and Goldthorpe 1992) interacts with other socio-demographic characteristics which, combined, seem to depict a subaltern class world characterized by its subordination in the division of labor and in political and social relations, but also by the shared certainty of not being or no longer being inscribed in the grand narrative of social mobility, be it for them or their children.

We systematically compare our results to INSEE’s data at the departmental level that we use as a kind of control group. By situating ourselves on this scale of comparison, we avoid drawing hasty conclusions about the under- or over-representation of this or that category among the Yellow Vests activists, which could simply be a reflection of the socio-economic fabric of the department. In doing so, we also show how difficult it is to question the demographics of the YV groups without placing them in their local configuration.

Table 2 below shows that compared to the department's population, the interviewed YVs are distinguished first of all by a concentration in the lowest SPCs with 59 people (41% of the group), i.e. + 15 points.^5^ Intermediate categories are also over-represented among the YVs, with 45 people (31%), i.e. + 13 points compared to the department. In terms of employment, the active population of the department is 50% compared to 76% among the YVs, i.e. + 26 points. However, among those in the labor force, the number of unemployed YVs is twice as high as at the department level (19% vs. 10%).

**Table 2:** Population 15 years or older by socio-professional category (aggregated level)

	District household	District household %	LHC YVs	LHC YVs %
SPC +	56,944	6	6	4
SPC intermediate	164,146	18	45	31
SPC-	236,175	26	59	41
Retired	293,664	33	30	21
Other inactive, never employed, students	149,939	17	4	3
Total	900,868	100	144	100

**Fig. 1:**
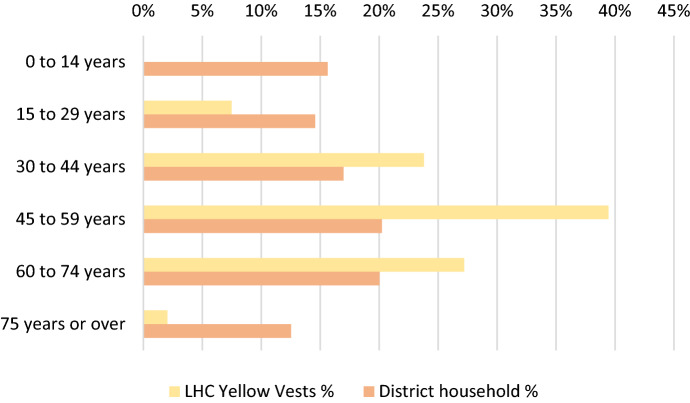
Population by major age group in 2018

This situation is partly explained by the age distribution, since as shown in Fig. 1, the proportion of people of working age among the YVs is much higher (70%) than at the department level (52%), mainly because of the scarcity in the movement of people aged 15–29, and at the other end of the life cycle, people aged over 75.

Table 3 compares the department’s population with our respondents by SPC. Three elements stand out. Firstly, craftsmen, shopkeepers and company managers are slightly more numerous among our respondents than in the department's population (+ 3 points), but above all, the latter are all heads of companies with fewer than ten employees, which places them on the lower side of the occupational scale. Secondly, contrary to the manual workers, the clerks are largely overrepresented among the YVs (+ 15 points). In fact, clerks and office workers have overtaken manual workers in France since 1993, in line with the development of services and the decrease of industrial employment. This is particularly marked in the department studied, where nearly 40% of salaried positions are concentrated in public services, mainly in health and social services, and 17% in retail trade activities. However, it should be noted that among the YV clerks, nearly two thirds work in the private sector as sales assistants, cashiers, self-service attendants, salespeople, and nearly one third work in the health and social sector as auxiliary staff in the services to individuals, especially home care, childcare, housekeeping, employees in bars, restaurants and other places of entertainment. Only 4% are office workers in the public sector. According to our field observations, this is partly due to the systematic pressure and threats that local elected officials put on civil servants, who were numerous on the roundabouts during the first weeks of the movement. (i.e. the police and the military, but also for city hall office workers, workers in municipal workshops, parks and roads maintenance workers, and garbage collectors) Finally, the intermediate professions are also over-represented in our group of YVs (+ 8 points compared to the department), and are concentrated in roughly equal proportions in the socio-educational, health and social work sector and in the commercial sector.

**Table 3:** Population 15 years or older by socio-professional category

	District household	District household %	LHC YVs	LHC YVs %
Managers and higher professions	56,944	6	6	4
Farmers	3391	0	3	2
Independent workers	45,756	5	12	8
Intermediary occupations	114,999	13	30	21
Clerks and office workers	153,281	17	46	32
Manual workers	82,894	9	13	9
Retired	293,664	33	30	21
Other inactive, never employed, students	149,939	17	4	3
Total	900,868	100	144	100

All in all, when we look in detail at the content of the categories of craftsmen and small businessmen, intermediate professions, clerks and manual workers, we can better understand what makes up, links or distinguishes the occupations most present on the roundabouts. Our first analysis shows on the one hand that the distinction between blue-collar and white-collar workers no longer makes much sense, and on the other hand that, far from constituting the beginning of a process of averaging of social conditions, the growth of the white-collar group, on the contrary, gives full meaning to the concept of a subaltern class.

The proximity between blue and white collars also stems from the similarity of jobs and the interweaving of tasks, as shown by the INSEE nomenclature. As Alain Chenu (1990) reminds us, depending on the status of their employer, cleaners may be classified as clerks (in private households) or as manual workers (in companies). Clerks and manual workers have almost similar incomes, live in the same neighborhoods, rarely own their homes, occupy more or less the same place in the social hierarchy, and form the most widespread type of household in France.

In Var, because of the economic fabric's structuration around tourism and local services, these low-skilled professions have grown significantly. They form a new popular constellation dedicated to serving the customer and the consumer, what Karjanen (2016) calls a “servant class.” With the emergence of new care services, this servant class is in large proportion composed by women. They represent 87% of the employees in this sector, but also most of the unqualified personnel working in retirement homes and hospitals, and home care assistants. Thus, whether in retirement homes or in the home hospitalization sector, the aging of the population is accelerating the formation of a proletariat in the so-called silver economy, which is at the heart of the YVs movement in the department studied. This trend is particularly marked in a department where population growth is increasingly due to net migration of retired people rather than to natural increase.

All in all, we are dealing with a vast social milieu that brings together low-income workers, the working poor or those on the verge of poverty, who exercise their profession essentially in the private sector. This social positioning can only give rise to a powerful subjective malaise and pessimism (Jeanpierre 2019), the effects of which are known on political behavior: electoral abstention and a marked withdrawal from political, union and association activities. (Im et al. 2019).

Here, the idea that material conditions of existence determine the individual consciousness by producing a set of collective representations, what Karl Marx calls ideology (see *The German Ideology*, 1846) is key. It invites to link a great proximity of material situation to common life styles. These lifestyles are embodied in the frequentation of the same living spaces, whether it be housing (distance from urban centers, type of housing), shops or places of leisure and relaxation. Moreover, the fifty or so life stories we conducted with YVs underline a growing feeling of isolation linked to the territorial withdrawal of public services since the 1990s, but also due to the increasing difficulties experienced in ensuring mobility because of new regulations on speed limits and increasing fuel prices.^6^

The lack of mobility also affects social mobility, which is largely stagnant and sometimes declining for the YVs we surveyed,^7^ a situation that interviewees most often associate with their concern about the impeded social mobility of their children, given the acceleration of social segregation in schools over the last thirty years. All this confirms their feeling of being left by the wayside and of having become invisible, supernumerary, hence the expression of one of our interviewees (Elisabeth, see below): “being considered as garbage and not as human beings”.

We will conclude with an element that reinforces the idea of a proximity of social status between YV’s activists, and of a community of experience around economic hardships, as well as of an increasing distance from the fate of the small and middle intellectual or business-focused bourgeoisies. A dimension that certainly was key to the success of the emergence of an “us feeling” among Var YVs, through a process of accelerated “identization.” (Melucci 1996).

**Fig. 2:**
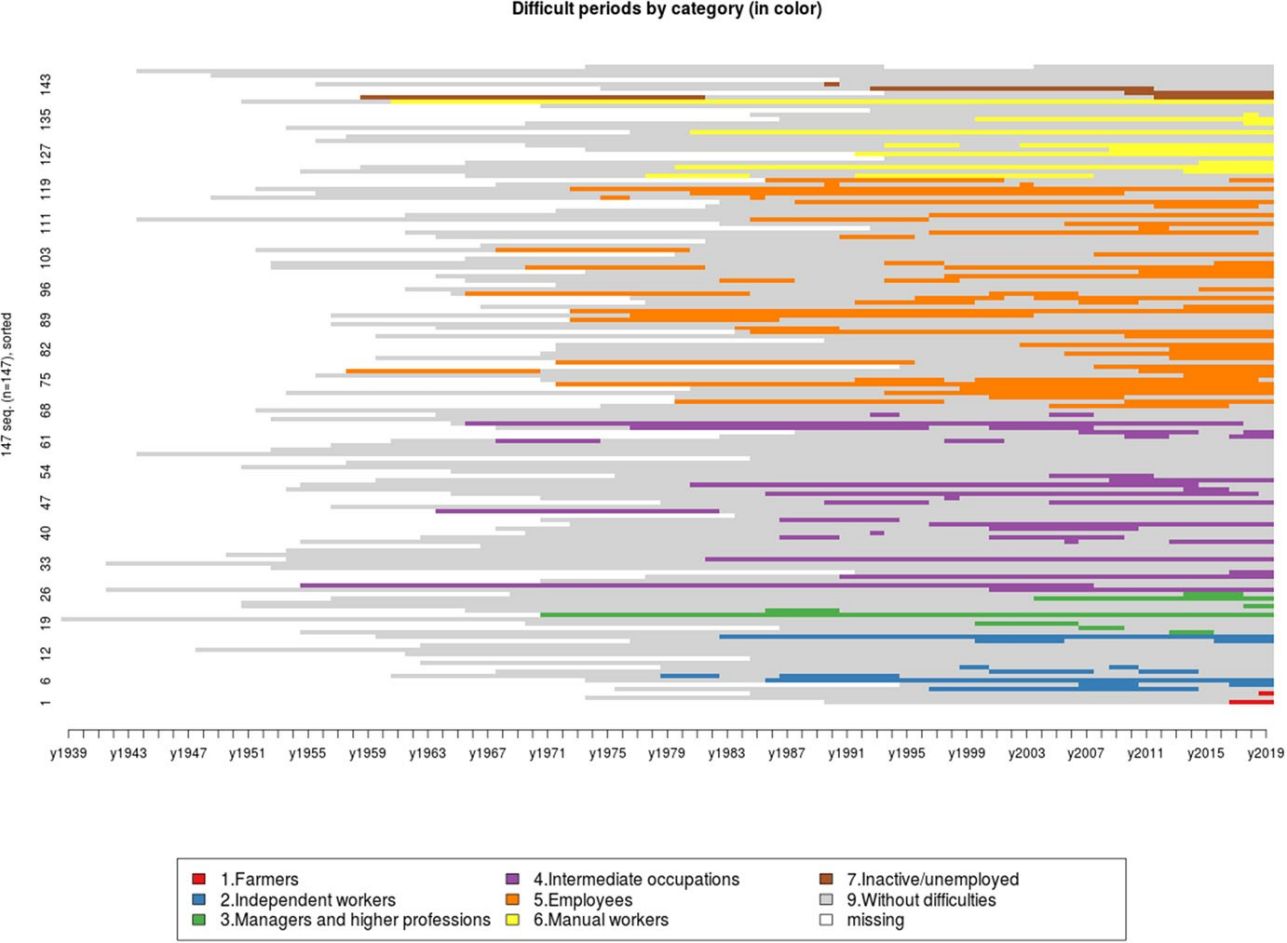
Reported difficulty periods by category

**Fig. 3:**
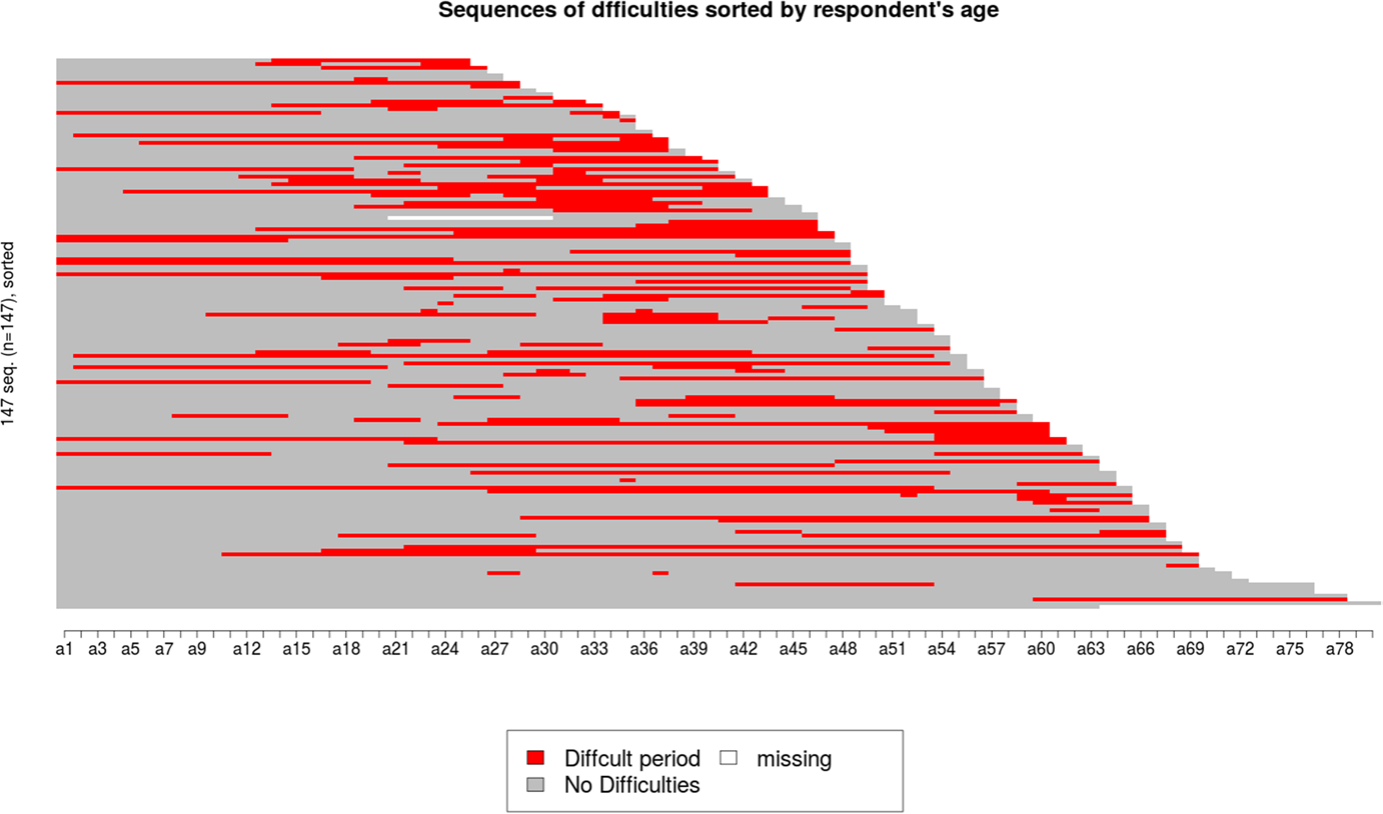
Sequences of difficulties by age

Figure 2 below proposes a sequential treatment of the periods of significant economic hardship mentioned by our interviewees along time. The result is striking: whatever the age of the people concerned, we can see that for the intermediate professions, blue and white-collar workers as well as the small self-employed, the times when it is no longer possible to make ends meet are concentrated around the period 2006–2018, even before the 2008 financial crisis, which, moreover, will have had a lasting effect on the least well-off categories. The sequential analysis also reveals that these episodes do not necessarily take place at the same time in their life course, as shown in Fig. 3. While some people grew up in very precarious situations and have all along their lives experienced periods of economic hardship, others have seen their lives stabilize after a difficult childhood, while some grew up in a stable environment with material comfort before experiencing major difficulties at work, in their affective life or in their health. But in the end, the vast majority have experienced hard times.

To better incarnate the different moments during which periods of economic hardship occur over the life course, we present here three examples of life paths in a concise manner. Elisabeth is a woman from a middle-class background. Born in 1955 and raised in the Paris region, her father was a veterinarian for the French Ministry of Agriculture and her mother was a factory worker and then an employee in a movie theater. At age 38, after her second marriage and a professional career as an insurance sales representative, she becomes chief administrative officer in her husband’s real estate company. 10 years later, her husband dies and the  hard times begin. Forced to close the business, she loses her job and finds herself alone to raise her second child, without the possibility of receiving unemployment benefits until 2006. Struggling to get a job, the economic difficulties follow one after another until she can no longer face them. At age 58, after ten years of economic hardship, she is forced to limit her budget to basic needs (food, housing) and to stop spending money on leisure activities. She also put health and household expenses (furniture, car) on hold and is forced to sell the single-family home she owned. She decides to return to Var, to live in a cabin on a piece of farmland that her husband owned. When Elisabeth joins the YV movement in 2018, she has a very small pension, raises her son alone, and has difficulty making ends meet.

Marc also comes from a rather comfortable background economically speaking, but very quickly experienced periods of economic hardship. He was born in 1990 and his father and mother, after their retirement as police officers, started a second professional career, his father as a company manager in the event industry and his mother as a childcare assistant. At the age of 18, he obtains a vocational training certificate in sales and at the age of 20 a professional degree in sales. The same year, he starts working in a supermarket as a salesman before moving on to become an employee in a fast-food chain. When, at the age of 24, he moves in with his partner and welcomes his first child, the difficulties begin. His job and his wife’s position as an interim health care worker allow them to survive more than to actually live. Things get complicated in 2018 when he quits his position and takes up a temporary job as a logistician in a supermarket. The expenses of the couple are then reduced to the bare essentials. In this context, he occasionally receives financial support from his relatives. When Marc joins the YV movement in 2018, he is going through a complicated period professionally and financially, a period that started very early in his life and that contrasts strongly with his parents’ life.

In contrast to Marc and Elisabeth, Sabine, who was born in 1956, grew up in a very underprivileged environment in the Lyon area. Her father had no professional activity and died when she was still a child, while her mother was a waitress in a school canteen. From the age of 18, she works a series of odd jobs as a factory worker, housekeeper, real estate hostess, cashier… After ten years of this, in 1983, she trains in dressmaking and becomes a costume designer. In 1987, she becomes a line cook after graduating from professional cooking school. In 2004, at the age of 48, she returns to studying and gets a diploma granting access to graduate studies which enables her to obtain a master’s degree and become a trainer and then a teacher, as a civil servant. She declares that she had financial difficulties throughout her life, from a very young age and until 2008, when she stabilized her situation by acquiring a job in the public sector. Her economic hardships ranged from restrictions on basic needs, to credit card debt, to giving up on major life plans. When Sabine joins the YV movement in 2018, she is in a comfortable situation but she immediately feels a surge of solidarity with the people she meets on the roundabout where she goes on November 17, deciding then to stay there on behalf of this community of experience of social suffering.

As these portraits show, experiences of economic hardship and social marginalization may have been a very strong glue in the continuous process of “boundary work” (Wimmer 2008) or “identity work” (Reger et al. 2008) who were deployed on the roundabouts and in the YV rallies.

### A localized and dynamic analysis of claims and worldviews

The proximity of social status and the common experiences of economic hardship can trigger anger, but are not sufficient to explain what drives mobilization and the feeling of sharing common outrages. It is then necessary to focus on the worldviews that the YV movement locally carries and its capacity to mobilize a plurality of individuals by politicizing their frustrations in the form of political consciousness. This is particularly important in the context of a non-categorical identity movement that cannot rely on pre-existing identities. Due to the duration of the movement, its complex evolution, and its local particularities, studying worldviews of the YVs is particularly challenging. Several studies have shown how YVs conversations can be compartmentalized into discrete topics (Boyer et al. 2020; Sebbah et al. 2019). They show congruent results. Mostly, the topics pertain to the organization of collective action, the formulation of claims, the denouncing of police violence, support and sympathy for the movement, and anger aimed at politicians. The YVs movement’s claims have been noted to have quickly broadened, from the narrow opposition to a fuel tax hike to the wider rejection of economic inequality as well as challenging of the ruling elite’s impunity. Although these studies analyze the relative weight of each claim at the time of data collection, they do not allow us to measure the evolutions, which is the only way to adequately describe the supposed broadening of the movement's claims. Also, they do not always specify the type of activists interviewed and relationships between claims and contextual events.

In this context, FB data helps to reconstruct reactions to local as well as national events from the “digital traces” left by the users. This type of source frees oneself from retrospective illusions and post hoc reasoning and allows to restore, as events have unfolded, the feelings expressed via status updates and comments. Second, the bulk of the text posts is used to create a corpus to be analyzed via automated text analysis. All in all, this enables us to restore the ways YVs have locally advanced their claims over time, some of them losing centrality in favor of others.

We collected data from the 6 active Facebook groups and pages that were linked to the roundabouts under study in the period from October 2018 to December 2019. We collected 56,954 posts, comments and answers, of which 34,109 contained text and could be analyzed for the purpose of this study. Because we were interested in the discourse co-constructed in the interaction between users, we pooled all comments and answers by the post they referred to, reconstructing discourse threads (*n* = 6339) and then jointly applied topic modeling and content analysis.

**Fig. 4:**
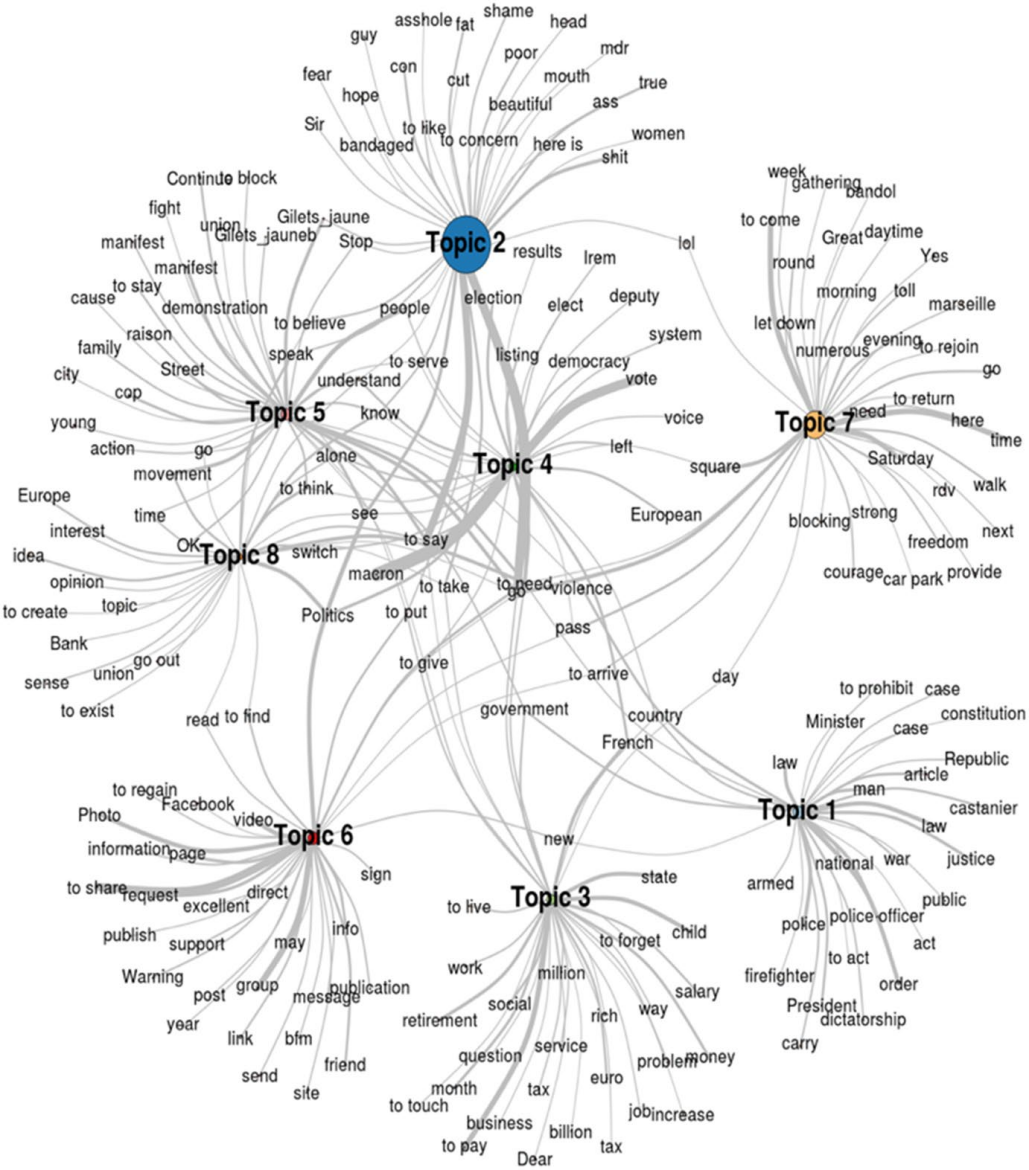
Graphical representation of topics and their most relevant words. *Note*: The wider the edge the higher the frequency in the topic; the larger the vertex the higher the topic distribution in the corpus

Figure 4 shows the 8 topics found in the configuration that gives the best solution in terms of triangulation of statistical fit indexes and interpretability of the results. We can now interpret the topics’ contents according to their most relevant words. The first topic deals with abuse of rights, such as the prohibition to demonstrate, with power abuse, discussing cases of politicians spending public money on expensive dinners, or with police violence. The second topic contains insults and expressions of anger aimed at the previously cited actors. The third one deals with the various demands driving the movement to reduce income inequality as well as social and political injustice. Conversations in topic 4 are focused on party politics and elections. Topic 5 debated the collective identity of the movement, such as who supports or opposes it, whether it should join forces with other protesters, and how to characterize its participants. Topic 6 deals with information sharing in the movement’s online spaces, especially people requesting collaboration to spread information or outrage. The seventh topic focused mainly on the organization of protest actions and meetings. Finally, topic 8 contains debates about various policies at the national and international level, such as national debt and whether France should break off of the EU.

Next, to investigate the development of themes over time and their relationship with contextual events, we pooled the threads by activist groups and weeks, following the weekly rhythm of the movement and keeping the specificity of each group. We then obtain time series which reveal the distribution of each topic across time.

Let’s start with the “social claims” evolution along time. It follows a clear upward trend, a phenomenon that has been widely linked in the case in point to a disorderly expansion of the level, nature, and range of demands, hence the ease with which the YVs’ claims could be so easily dismissed as irrational.

**Fig. 5:**
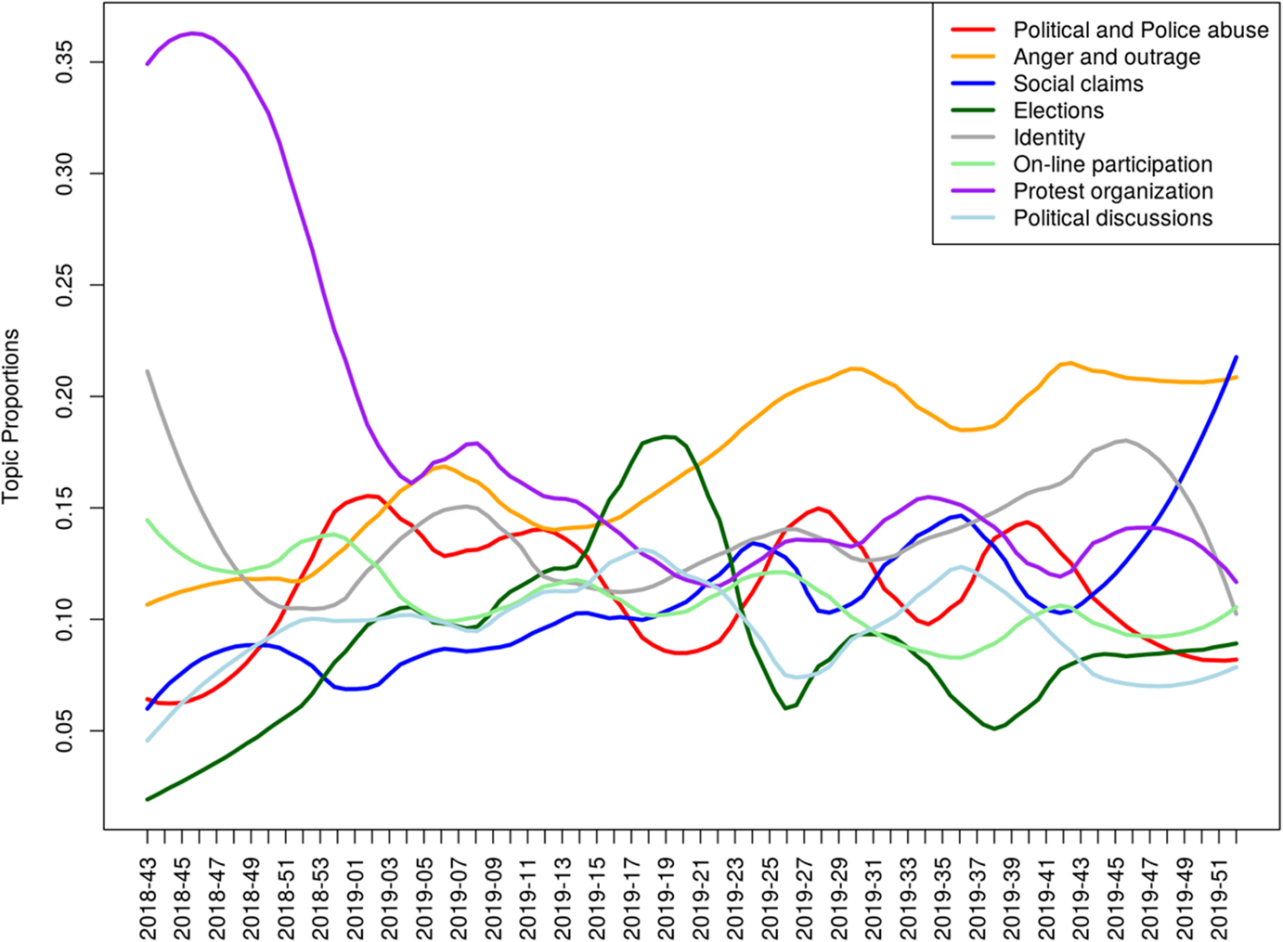
Distribution of topics across time. *Note*: Time is expressed on the x axis in a year-week format, starting with the 43^rd^ week of the year 2018

**Fig. 6:**
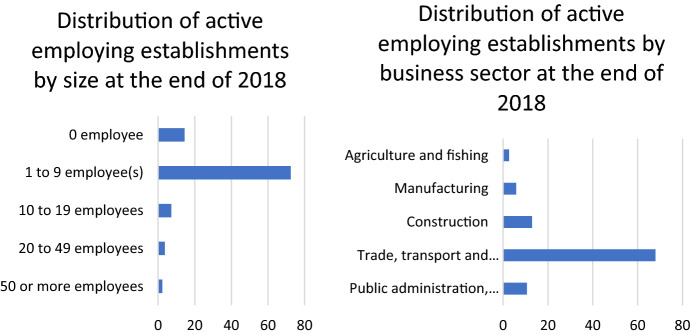
Distribution of active employing establishments by sector of activity and size at the end of 2018 (Insee)

However, the YV movement, when examined closely, falls within a long succession of anti-tax movements which are typical examples of situations when the working class directly speaks up in political arenas from which it is generally excluded (Delalande and Spire 2010). From this perspective, we may not have sufficiently built on this conclusion of Norbert Elias, which sees individual and collective resistance to taxes at the very heart of a dynamic process in our modern societies. Indeed, *“’grants’ and taxes are the mirror image of the interdependence of social groups and of the power relations which govern this”* (Elias 1982, 153) Because it symbolizes inclusion in a national community, the payment of taxes de facto leads to a collection of individual rights and claims on this community. This is undoubtedly the reason why, in the studied groups, work-related demands addressed to employers are very rare, in favor of questioning the political authorities, who are considered essentially responsible for their malaise. If this is to be related to the rejection of intermediary bodies, it is also, if not primarily, the product of a logic of bypassing the classic mechanisms of collective bargaining for people whose status as “salaried workers” is no longer self-evident (Fig. 5). This echoes a double dynamic of “dispersion and polarization” linked to the explosion of precarious employment and, on the other hand, a process of “re-proletarianization” linked to the unraveling of social protections and “disaffiliation” (Béroud et al. 2016; Castel 1995). This phenomenon is all the more marked in that, in the region studied, around 70% of the employers’ establishments are concentrated in the trade, transport and various personal services sectors companies and have less than 10 employees, (See Fig. 6 below). In fact, most of the YVs in the department are caught up in “work logics” (e.g. Kitschelt and Rehm 2014) that keep them far from the classical image of the factory worker. For the most part employees of very small companies, they maintain a close relationship with their employers, whose economic difficulties they know.

The “police abuse” topic follows the prevalence of this topic in the news media. It starts picking up after the fourth week (2019–50), where the government started committing to a strategy of heavy police and judicial repression, that will escalate again after the degradations of the Champs-Elysées on March 16th (2019–13), and the appointment of a hardline police prefect in Paris, before coming back again sporadically when police abuse makes the news. It should be noted that the phases of increasing criticism of repressive action by the state seem to be reflected in a concomitant decrease in social claims, which indicates that the two topics concentrate the rejection of the government, the people who run it, and the policies it implements.

The “anger and outrage” topic is following a clear upward trend, then dominating all the others in volume (2019–2019). Gathering the violent interpellations of power, insults and what we call “secessionist” remarks, the pace of this topic clearly indicates a process of continuous political radicalization, which neither the concessions of the government nor the fierce repression are enough to contain. The case is not so rare and the literature has studied how situations where activism is repressed or criminalized can be a powerful lever for transformation of people’s personal identity, hence politicizing the resulting social identification. (Combes and Fillieule 2011 for a review).

Within the groups we study, one notes that this topic becomes dominant after the disappointment of the European elections. Indeed, the “elections” topic starts very low, showing a deep rejection. It then slowly rises as participants attempt to position themselves in relation to the May 2019 European elections, at which points it briefly peaks as the main topic discussed, before diving sharply again as the “anger and outrage” topic becomes dominant, which shows the anticlimactic letdown that this ballot was for movement participants.

Finally, the radicalization process here observed has to be related to two other phenomena suggested by our field observations. First of all, if participation in longstanding occupations of roundabouts contributed the most to insure the duration and extension of the movement, it is also because it provided participants a new “plausibility structure” (Berger and Luckmann 1966) by elevating roundabouts to the status of an agora in which small groups are formed, prompted by discussions and an influx of onlookers, drawn by the good-natured atmosphere of this unprecedented event that their spatial routines led them to approach. What struck the observer was the freedom of speech, the feeling that it was alright to “say anything,” about oneself and one’s situation, to "question everything.” In this sense, the physical occupation of a roundabout is not only a citizen (re)appropriation, similar on a smaller scale to media images of the Indignados in Spain or Occupy in the US (Ancelovici et al. 2016). The transgressive occupation materializes and symbolizes the presence, by force, of disadvantaged groups in the public space and is a visible manifestation of their claim to weigh in on the course of history. Thus, they come to construct a free space within which individuals regain a form of autonomy, partially sheltered from looks and intrusions from “the outside.” All this has nurtured the radicalization process, as beliefs are reinforced through strong affective bonds within the in-group while the time-consuming nature of activism puts a strain on relationships outside of the movement.

Another possible complementary explanation for this radicalization process refers to the fact that the number of YVs in the area studied, as elsewhere in France, has been steadily decreasing since after winter 2019. This suggests the hypothesis, largely verified by our field observations, that as time develops, the least committed participants would have slowly demobilized for various reasons, leaving only a small subset of YVs mobilized. Therefore, it could be that only core members of the groups remained active, for which such or such topic could have been more central than for other members that had dropped out. To control for this selection bias, we calculated the number of active users per week. Doing so, we do not observe a higher presence of the topic in the discussions in relation to a drop in the number of users (only core members remain). A Pearson partial correlation trend test (*r* = 0.26, *p* = 0.034), controlling for the number of users, confirmed our results.

Last but not least, the trends followed by topics referring to “protest organization,” “online participation,” and “political discussions” also suggest some hypotheses. Figure 6 shows that in the very first weeks of the mobilization, the immense majority of the exchanges concern the organization of the movement, its structuring and its networking, the relevant modes of action to implement. Like all movements without a prior mobilization structure, without a leader and without a program, the internet is the virtual structure that concretely allows to go from the spontaneous gatherings of November 17 to a movement with an identity and a set of strategic and ideological directions. But very quickly, as things were being put in place, the YVs were to give less and less space to these questions, only to come back to them, but to a lesser extent, when the disappointment of the European elections gave rise to lively debates on a possible electoral strategy for the next municipal and then regional elections.

Also, the maintenance of a regular level of discussions around the “identity” of the movement, but also “online participation” and “political discussions”, suggests that participation in Facebook groups plays the role of a low-intensity support and information network akin to an “abeyance structure” (Taylor 1989), meaning that it helps maintain continuity in the movement’s colder times and promotes remobilization through awareness-raising on new issues and calls for solidarity within the groups, as has been observed with the advent of protests over policies adopted in response to the Covid-19 crisis. This purpose of FB groups could be of critical importance to the persistence of mobilization in a “leaderless” movement devoid of formalized organizations.

This type of longitudinal data offers to refine our analysis much further than the static model allows. It shows how the different topics can be linked to specific time frames, how they compete and how the movement’s discourse structures itself across time. It is worth noting however that these data speak only of the local FB groups from the chapters we are studying. It is therefore difficult to distinguish local and national trends in the discourse we analyze. Once the various layers of agenda-related surges have been explained, it is nonetheless possible to use topic proportions to investigate a particular group’s “ambience”, for example whether it favors direct action or more deliberative practices.

## Conclusion

In this article, we show how an innovative localized and longitudinal analysis can restore time and place as crucial variables in understanding how the YV movement has been constructed and reconstructed in a constant transformative flux. By studying both social positions and claims-making within a specific local setting over time, we find that the Var YVs constitute a subaltern class that de-singularizes individual hardship.

Furthermore, we argue that the YVs are not in favor of reducing government intervention but rather demand better state support for their social and economic safety and criticize its disintegration by neoliberal policies that benefit only the wealthy. We have equally pointed out that the participants’ worldviews are very diverse but converge over time towards so-called secessionist worldviews.

Confronted by police repression, government disregard and mistreatment, and the resignation of less engaged activists, the remaining participants form new and radicalized affinities. The continuation of collective action shapes and politicizes a range of social positions further into a more clearcut political consciousness. We argue that this is a process of political radicalization, reflected in demands that move from a discourse of negotiation to calls for profound change in the functioning of institutions and the redistribution of wealth. In other words, the YV upsurge from December 2018 until January 2019 did not result in a traditional protest movement but evolved into a secessionist movement that turned its back on power, elites and more generally on experts of all kinds.

Results from our investigation of the YVs in the Var region partially confirm previous research on the YV movement. However, the localized and longitudinal method applied implies that we cannot generalize our findings easily, though it offers a more in-depth understanding. Several studies present interpretations of the movement as populist, while others argue that it is class struggle developed into protests for social injustice and moral economy. Similarly to Guerra et al.’s (2019) definition of the YV movement as economic populism, we note the use of virtue and economic utility as opposing principles between “us, the common people” and “the elites.” However, the YVs in the Var do not target all elites, only those who “are undeserving of” their high position in society because they monopolize key cultural resources to position themselves advantageously in society. This conceptualization became particularly recurrent on the roundabouts and in demonstrations in the aftermaths of the great national debate and goes along a process of “political awakening” that many of our informants experience.

Research from the US, which takes into consideration time and place, can help us understand the politicization processes of the YVs in the Var. In her ethnography of folks in rural Wisconsin, Cramer (2016) observes the processual creation of a “rural consciousness”. Understood as an identification with a place and a social group shaped by sentiments of distributive injustice and relative deprivation, this notion displays the importance of place and class identity construction in people’s political sense-making. Further, the secessionist dimension of the YVs’ politicization and solidarity ties between class fractions can be interpreted as a form of “active citizenship” (Braunstein 2017), i.e. a common way of thinking, mode of action, and relation to participatory democracy that fuses political vigilance with personal virtue. In *Prophets and Patriots*, Braunstein (2017) develops the concept to capture the common political attitudes of two politically opposing groups: a faith-based progressive community-organizing coalition (the Prophets) and a Tea Party chapter (the Patriots). Despite their differences, the two groups share similar concerns about disparities between elites and ordinary Americans, starting with a belief that the economy serves a few at the expense of the many and that ordinary people are not being included in decisions about how to achieve the productive, healthy, and comfortable lives they had been promised. By shifting the focus from ends to means, and from specific policy preferences to concerns about the political process, Braunstein can help explain what unifies groups with opposing political positions. Similarly, the YVs adopt a vigilant stance resulting from political involvement without being personally engaged in institutional politics. This posture marks an opposition to a “deferential” form of citizenship characterized by trust in government, experts and elites.

These suggestions drawn from the US literature are an invitation to investigate how social consciousness driven by solidarity and active citizenship can help us understand the evolving claims and worldviews of the YVs in a context marked by the COVID-19 crisis and a significant participation of many of them in the mobilizations against masks, vaccines and the Covid-19 certificate.

## Footnotes

1 This work is part of a research funded by the Swiss National Research Fund (MYF project—The Mystery of the Yellow France). We warmly thank Eli Melby and the two anonymous reviewers of the first version of this text for the improvements they made to it.

2 According to our field observations, if at the very beginning of the movement, part of the YVs were not having a FB account nor were familiar with social networks, this changed dramatically and fast, in connection with the growing rejection of traditional information channels and the fact that the life of the roundabout clusters was shown on FB, both in its festive and activist aspects.

3 The lengthy efforts made here to achieve relative significance are in line with previous sampling strategies that have been recognized as successful in constructing meaningful samples despite the absence of a reference population. (see Masclet 2014; Fillieule and al. 2018).

4 Which means on one side that the work is still ongoing and the final number of LHC is not yet attained. But also that a very few number of people did refuse to fill it. For most of those who were reluctant, it took time (up to two years in one case!) to convince them to participate, but the long-term presence on the roundabouts, and our active participation to many protest events have removed almost all obstacles. less than 5% of the requested persons have opposed a definitive refusal.

5 The percentages here are indicative only and are intended to facilitate comparison with data on the population of the department under study.

6 One should note that dependence to travel by car is also to be related to the scarcity and disorganization of public transport in this semi-rural department as soon as one moves away from the coast.

7 We constructed mobility tables based on the LHCs and the occupations of both parents per household. The low N makes a fine interpretation difficult, so we do not produce it at this stage, but clearly the dominant trend is one of stagnant or downward mobility.
